# Quantification of rare somatic single nucleotide variants by droplet digital PCR using SuperSelective primers

**DOI:** 10.1038/s41598-023-39874-0

**Published:** 2023-11-03

**Authors:** Verónica Pablo-Fontecha, Eva Hernández-Illán, Andrea Reparaz, Elena Asensio, Jordi Morata, Raúl Tonda, Sara Lahoz, Carolina Parra, Juan José Lozano, Anabel García-Heredia, Alejandro Martínez-Roca, Sergi Beltran, Francesc Balaguer, Rodrigo Jover, Antoni Castells, Ramon Trullàs, Petar Podlesniy, Jordi Camps

**Affiliations:** 1 Translational Colorectal Cancer Genomics, Gastrointestinal and Pancreatic Oncology Team, Institut D’Investigacions Biomèdiques August Pi i Sunyer (IDIBAPS), Hospital Clínic de Barcelona, Rosselló 149-153, 4th Floor, 08036 Barcelona, Spain; 2 Centro de Investigación Biomédica en Red sobre Enfermedades Neurodegenerativas (CIBERNED), 28029 Madrid, Spain; 3 CNAG-CRG, Centre for Genomic Regulation (CRG), Barcelona Institute of Science and Technology (BIST), 08028 Barcelona, Spain; 4 Centro de Investigación Biomédica en Red de Enfermedades Hepáticas y Digestivas (CIBEREHD), 28029 Madrid, Spain; 5 Servicio de Medicina Digestiva, Hospital General Universitario de Alicante, Instituto de Investigación Sanitaria y Biomédica de Alicante (ISABIAL), 03010 Alicante, Spain; 6 Present Address: Neurobiology Unit, Institut d’Investigacions Biomèdiques de Barcelona (IIBB-CSIC), Institut D’Investigacions Biomèdiques August Pi i Sunyer (IDIBAPS), 08036 Barcelona, Spain; 7 Unitat de Biologia Cel·lular i Genètica Mèdica, Departament de Biologia Cel·lular, Fisiologia i Immunologia, Facultat de Medicina, Universitat Autònoma de Barcelona, 08193 Bellaterra, Spain

**Keywords:** Genotyping and haplotyping, PCR-based techniques, Next-generation sequencing, DNA sequencing, Colorectal cancer

## Abstract

Somatic single-nucleotide variants (SNVs) occur every time a cell divides, appearing even in healthy tissues at low frequencies. These mutations may accumulate as neutral variants during aging, or eventually, promote the development of neoplasia. Here, we present the SP-ddPCR, a droplet digital PCR (ddPCR) based approach that utilizes customized SuperSelective primers aiming at quantifying the proportion of rare SNVs. For that purpose, we selected five potentially pathogenic variants identified by whole-exome sequencing (WES) occurring at low variant allele frequency (VAF) in at-risk colon healthy mucosa of patients diagnosed with colorectal cancer or advanced adenoma. Additionally, two *APC* SNVs detected in two cancer lesions were added to the study for WES-VAF validation. SuperSelective primers were designed to quantify SNVs at low VAFs both in silico and in clinical samples. In addition to the two *APC* SNVs in colonic lesions, SP-ddPCR confirmed the presence of three out of five selected SNVs in the normal colonic mucosa with allelic frequencies ≤ 5%. Moreover, SP-ddPCR showed the presence of two potentially pathogenic variants in the distal normal mucosa of patients with colorectal carcinoma. In summary, SP-ddPCR offers a rapid and feasible methodology to validate next-generation sequencing data and accurately quantify rare SNVs, thus providing a potential tool for diagnosis and stratification of at-risk patients based on their mutational profiling.

## Introduction

Tracking driver mutations is essential for patient management. Nevertheless, the methodology to detect low-represented single-nucleotide variants (SNVs) have yet to be standardized. Next-generation sequencing (NGS) approaches not only allow the identification of novel driver events^1^, but also reveal that mutations in genes related to cancer may accumulate in healthy cells without conferring a malignant state^2^. This raises questions about the functional role of these mutations as universal biomarkers. Despite NGS has been already utilized for molecular diagnosis and prognosis^3,4^, its usage to address personalized medicine in oncology remains under debate. Technical difficulties, such as the detection of mutations present in a small number of cells, the amount and quality of DNA, the coverage of each variant, and the total number of reads, have limited the insight into its applicability. Different strategies have been developed to increase the sensitivity to identify pathogenic mutations^5–7^; however, they are costly and time-consuming. Additionally, although NGS at standard coverages has enabled the detection of common variants across cancers, it may also require large cohorts and/or subsequent orthogonal validations to assure that mutations are genuine and not technical artifacts^8^. Alternatively, NGS at low coverage (< 20x) is usually performed^9^, but it might be insufficient to detect somatic mutations at low variant allele frequencies (VAF)^10^.

Polymerase chain reaction (PCR)-based methods represent the surrogate and complementary assays to NGS. Among them, droplet digital PCR (ddPCR) stands out by its sensitivity and precise quantification. ddPCR measures an absolute count of target DNA copies per sample without a standard reference, providing the chance to quantify somatic variants at rare frequencies. This approach has been already proven to be effective when utilizing specific hydrolysis probes^11,12^. Nevertheless, the use of these fluorogenic probes, in which the discriminative power between targets is based on a differential labeling for the wild-type and mutant alleles, requires the design and synthesis of two sequence-specific oligonucleotides that recognize each allele and detecting fluorescence in two different optical channels^11,12^. The design and optimization of this assay might be laborious and costly, compromising its implementation in the clinical setting. Thus, as frequent genetic testing is desirable for patient management, there is an unmet need to identify a cost-effective, sensitive, and highly reliable method.

SuperSelective primers, which enable the amplification of SNVs in the presence of an excess of the corresponding wild-type target, consist of a relatively long 5'-“anchor” sequence (average of 20 nucleotides) that strongly hybridizes to target DNA fragments, and a very short 3'-“foot” sequence containing the interrogated nucleotide that targets the site of the somatic variant. A “bridge” sequence between the “anchor” and the “foot”, which does not hybridize with the target sequence, provides a single-stranded bubble that contributes to the selective amplification of the SNV^13^. It has been previously reported that using regular primers, while slowing PCR kinetics, does not allow the differentiation of single nucleotide mismatches^14,15^. However, the combination of a high-binding affinity segment (5’-“anchor”) and an unstable short region (3’-“foot”) provides selectivity to amplify the target sequence by standard intercalating dye chemistry detection. SuperSelective primers have been previously used in real-time PCR (qPCR) assays^13,16–18^. However, qPCR relies on the cycle of quantification for estimating the abundance of each target mutation. This relative quantification that is performed by the abundance of a reference gene, although useful for positive–negative discrimination analysis, might result in a biased quantification of the amount of relevant somatic variants.

In this study, we have designed and optimized the combination of SuperSelective primers and ddPCR to quantify rare low-frequency SNVs previously identified by NGS in both cancer and normal mucosa of colorectal specimens. The SP-ddPCR assay includes the synthesis of DNA control templates, the design of oligonucleotide sequences, and the optimization of the primers selectivity to amplify mutant alleles in human genomic DNA samples.

## Results

### Selection of pathogenic variants in normal colonic mucosa

The whole-exomes of 48 surrounding normal mucosae were sequenced at a mean coverage of 85x. Variant calling was performed for each individual sample and mapped against their matched peripheral blood leukocytes (PBLs), leading to the identification of multiple somatic mutations per patient (x̅ = 251 variants/advanced adenoma (AAD) lesion; x̅ = 109 variants/colorectal carcinoma (CRC) lesion).

The implementation of our criteria for SNV prioritization (see Materials and methods) resulted in the inclusion of five SNVs present in normal mucosa (two corresponding to patients with AAD and three to patients with CRC) as the best candidates to test our quantitative ddPCR method (Table 1). To note, *APC* mutations c.2626C>T and c.4128T>A occurring in CRC samples at VAF 42.96% and 6.35%, respectively, were selected to validate our design for VAF quantification.

**Table 1:** List of variants identified by whole-exome sequencing (WES) and prioritized for ddPCR validation in colorectal lesions and﻿ their surrounding normal mucosa from patients with CRC or AAD.

Chromosome	R^†^	A^‡^	Annotation	Annotation impact	Gene name	HGVS.c	COSMIC mutation ID	Total positive prediction tools
CRC variants								
5	T	A	Stop gained	HIGH	*APC*	c.4128T>A	COSM18861	5
5	C	T	Stop gained	HIGH	*APC*	c.2626C>T	COSM18852	5
CRC surrounding mucosa variants								
9	G	A	Missense	MODERATE	*LAMC3*	c.1241G>A	COSM1256308	3
14	G	A	Missense	MODERATE	*NRXN3*	c.1421G>A	COSM4973279	5
19	C	T	Missense	MODERATE	*ASNA1*	c.193C>T	COSM1680708	5
AAD surrounding mucosa variants								
9	G	A	Missense	MODERATE	*NTRK2*	c.220G>A	COSM1741429	5
8	C	T	Stop gained	MODERATE	*FABP4*	c.109G>A	COSM272654	4

^†^R, Reference.

^‡^A, Alternative.

### Sensitivity and selectivity of SuperSelective primers

**Figure 1:**
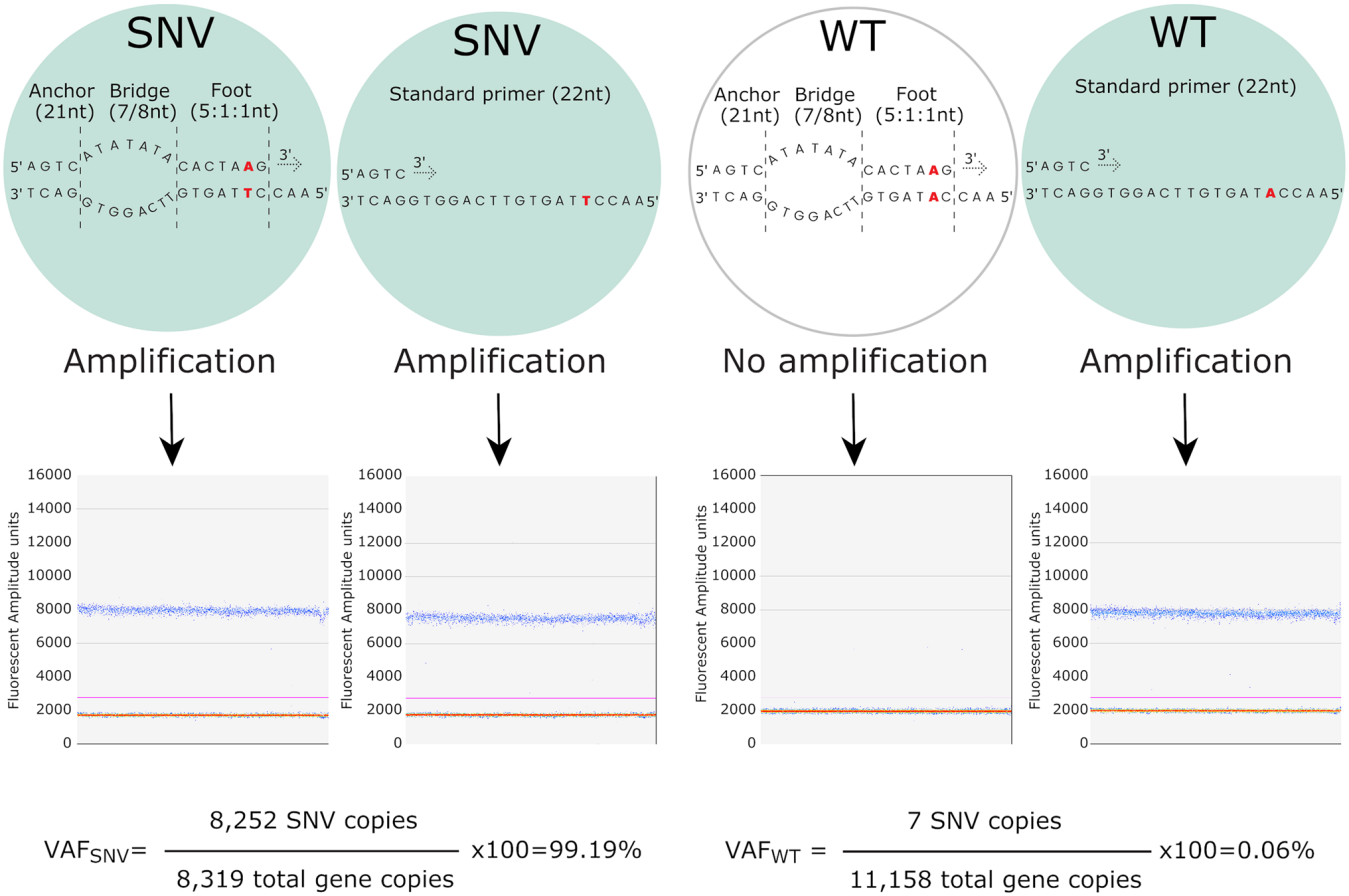
Diagram of the SP-ddPCR (SuperSelective primers in droplet digital PCR) optimization method to test SuperSelective primer accuracy and selective amplification of the allele of interest using DNA templates containing 10,000 molecules of the target loci bearing either the wild-type allele or the SNV. These measurements correspond to the estimation of 100% and 0% VAF by ddPCR. Suitable SuperSelective primers designed to detect *APC* c.4128T>A are shown as an example. WT, wild-type.

**Figure 2:**
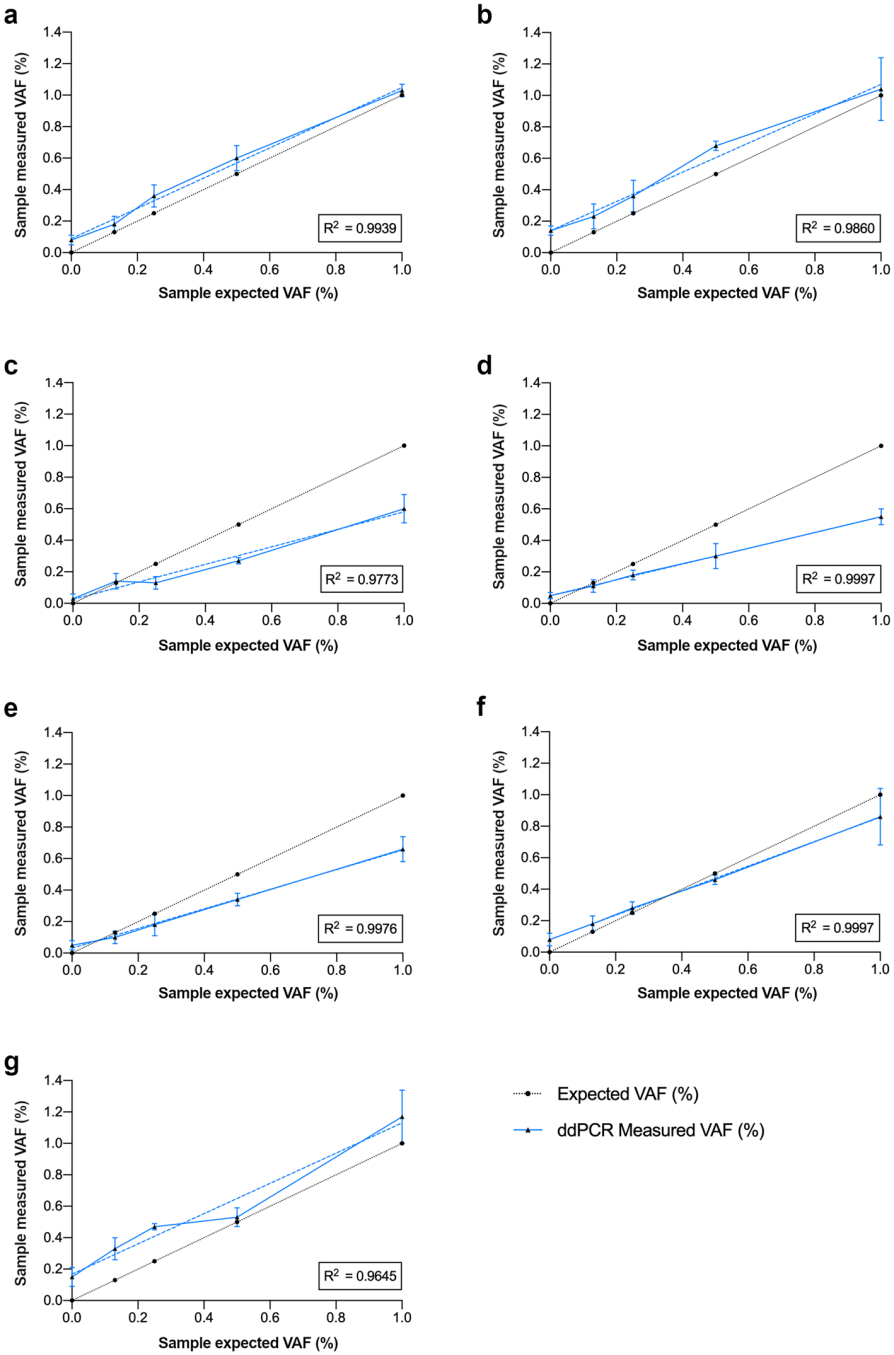
SP-ddPCR performance for quantification of rare SNVs measured in synthetic plasmid mixtures simulating 1.00, 0.50, 0.25, 0.13 and 0.00% VAFs. VAFs (%) measured by ddPCR are plotted versus expected theoretical VAF (%) for serial dilutions of plasmids containing the corresponding SNV sequence into the respective wild-type background consisting of plasmids containing the wild-type allele. Each mixture was measured in triplicate. Dots express mean values and error lines indicate standard deviations. R squared values of Pearson correlation between measured and expected VAF are displayed within each plot. (**a**) *APC* c.2626C>T, *p*-value 0.002 (**b**) *APC* c.4128T>A, *p*-value 0.0007 (**c**) *FABP4* c.105G>A, *p*-value 0.001 (**d**) *NTRK2* c.220G>A, *p*-value 0.000002 (**e**) *LAMC3* c.1241G>A, *p*-value 0.00005 (**f**) *NRXN3* c.1421G>A, *p*-value 0.000002 (**g**) *ASNA1* c.193C>T, *p*-value 0.003.

First, we identified an appropriate SuperSelective primer for quantifying each SNV by ddPCR using in silico samples with 100% and 0% VAF (Fig. 1, Supplementary Table 1). To calculate the ddPCR VAFs, the total number of target copies was quantified by standard primers. Such quantification was equivalent when up to four standard primers were tested (Supplementary Table 2, Supplementary Fig. 1). Next, selectivity of SuperSelective primers in ddPCR was analyzed with a serial dilution of each plasmid carrying the SNV mixed with the corresponding wild-type plasmid. Based on template concentrations from ddPCR reactions achieved with non-selective primers, sample mixtures were generated to simulate VAFs of 1.00, 0.50, 0.25, 0.13 and 0.00%. A total number of 10,000 molecules per 20 μl of PCR reaction were measured by ddPCR (n ≥ 3 for each mixture). All SuperSelective primers (Table 2) showed a linear quantification of increasing proportions of the SNV in a wild-type background (Pearson R squared ranging from 0.9997 to 0.9645, *p*-values ≤ 0.003) (Fig. 2a-g). Although expected VAFs were dependent on the target sequence and the primer design, the quantification of the expected 1% VAF ranged from 0.55 ± 0.05% to 1.17 ± 0.17% for the total number of mutant molecules of *NTRK2* and *ASNA1*, respectively. Contrastingly, when we compared SuperSelective with standard primers, the results revealed that three different standard primer designs were not able to discriminate the SNVs at *APC* c.2626C>T and *APC* c.4128T>A (Supplementary Table 3), confirming that one mismatch in a long regular primer is not sufficient to suppress the amplification of wild-type targets.

**Table 2:** Primer sequences utilized to quantify WES-derived VAFs by ddPCR.

Target gene (HGVS.c)	Primer sequence (5’-3’)		Amplicon sequence (5’-3’)	SuperSelective direction and strategy
*APC (*c.4128T>A*)*	S^†^	GTGCTCAGACACCCAAAAGTC -ATATATA-CACTAAG	GTGCTCAGACACCCAAAAGTCCACCTGAACACTA(T/A)GTTCAGGAGACCCCACTCATGTTTAGCAGAT	Forward; 21–7/8–5:1:1
	C^‡^	ATCTGCTAAACATGAGTGGGGTC		
	U^§^	GTGCTCAGACACCCAAAAGTC		
*APC (*c.2626C>T*)*	S^†^	GGCTGCAGTGGTGGAGAT- ATATATAT -CCTCAC	GCAACAGAAAATCCAGGAACTTCTTCAAAG(C/T)GAGGTTTGCAGATCTCCACCACTGCAGCC	Reverse; 18–8/7–4:1:1
	C^‡^	GCAACAGAAAATCCAGGAACTTCT		
	U^§^	GGCTGCAGTGGTGGAGAT		
*LAMC3 (*c.1241G>A*)*	S^†^	CACTGAGCGAGTGGAACCC-ATATATAT-ACAGTG	CCACGGTGACTGGCTGGAAGTGTGACC(G/A)CTGTCTGCCCGGGTTCCACTCGCTCAGTGAGG	Reverse; 19–8/6–4:1:1
	C^‡^	CGGTGACTGGCTGGAAGT		
	U^§^	CCTCACTGAGCGAGTGGAACCC		
*NRXN3 (*c.1421G>A*)*	S^†^	AACGTATGGGCTCCATCTCCT-ATATATAT-TTCC AC	AACGTATGGGCTCCATCTCCTTTGACTTCC(G/A)CACCACAGAGCCCAATGGCCTGA	Forward; 21–8/5–4:1:1
	C^‡^	TCAGGCCATTGGGCTCTGT		
	U^§^	AACGTATGGGCTCCATCTCCT		
*ASNA1 (*c.193C>T*)*	S^†^	GGGTCTGTGGAGATGATCAGAA-ATATATAT- TCACAC	CTGGCAGTCCAGCTCTCCAAGGGG(C/T)GTGAGAGTGTTCTGATCATCTCCACAGACCC	Reverse; 22–10/5–4:1:1
	C^‡^	CTGGCAGTCCAGCTCTCC		
	U^§^	GGGTCTGTGGAGATGATCAGAA		
*NTRK2 (*c.220G>A*)*	S^†^	CATCTTCGTTGATGATTTCTAACCTTT -TATATAT-GTTTGTG	CACTCTCTGCTTTGTTACAGTTTCATC(G/A)CAAACCAGAAAAGGTTAGAAATCATCAACGAAGA	Reverse; 28–6/4–5:1:1
	C^‡^	CACTCTCTGCTTTGTTACAGTTTCA		
	U^§^	TCTTCGTTGATGATTTCTAACCTTT		
*FABP4 (*c.109G>A*)*	S^†^	GCTTTGCCACCAGGAAAGTG-TATATAT-CATGAC	CACTGATGATCATGTTAGGTTTGG(C/T)CATGCCAGCCACTTTCCTGGTGGCAAAGC	Reverse; 20–6/5–4:1:1
	C^‡^	TCACACTGATGATCATGTTAGGTTTG		
	U^§^	TTTGCCACCAGGAAAGTG		

^†^S, SuperSelective primer.

^‡^C, usual primer used in combination with S or U.

^§^U, usual primer.

Sample mixtures simulating 0% VAF were used to establish a limit of detection (LOD) for each SuperSelective primer assay. LOD was defined as the average signal plus three times the standard deviation of nine or more ddPCR VAF measurements. Average LOD across assays was 0.18%. LODs obtained for each assay were 0.18% (0.08% + 3 · 0.03%) for *APC* c.2626C>T, 0.22% (0.14% + 3 · 0.03%) for *APC* c.4128T>A, 0.14% (0.05% + 3 · 0.03%) for *LAMC3* c.1241G>A, 0.32 (0.15% + 3 · 0.05%) for *ASNA1* c.193C>T, 0.19% (0.08% + 3 · 0.04%) for *NRXN3* c.1421G>A, 0.11% (0.05% + 3 · 0.02%) for *NTRK2* c.220G>A and 0.10% (0.03% + 3 · 0.03%) for *FABP4* c.109G>A. To ensure that LODs estimated with plasmid molecules were appropriate for high complexity human genomic samples, an average of 33 ng of commercially available human genome DNA was measured by SP-ddPCR (10,000 haploid genome equivalents per 20 μl reaction). ddPCR VAFs using the wild-type human genome were below LODs for *APC* c.2626C>T, *APC* c.4128T>A, *LAMC3*, *ASNA1*, *NRXN3* and *FABP4* assays. However, SuperSelective primer designed to quantify *NTRK2* c.220G>A showed a higher ddPCR VAF in wild-type human genomic background than the estimated LOD. Thus, LOD for this SNV quantification was recalculated using reference human genome to simulate 0.00% VAF and was expressed again as the average signal plus three times the standard deviation of nine or more ddPCR VAF measurements. Results showed a LOD of 0.40% (0.19% + 3 · 0.07%).

Similarly, sample mixtures were generated with synthetic molecules to simulate 100, 31.6, 10.0, 3.16, 1.00, and 0.00% VAFs. These samples were utilized as templates for VAF quantification both in qPCR and ddPCR using the corresponding primer sets. SuperSelective primers were able to detect the presence of the corresponding mutant allele not only in ddPCR but also in qPCR reactions ranging from 100% to 1.00% VAF. However, consistent throughout the seven assays performed, SuperSelective primers in ddPCR showed a lower deviation from the expected VAF compared to qPCR, which was based on cycle of quantification (Cq) values of total and mutant amplification (Supplementary Fig. 2a-g). In addition, for some variants, such as in *LAMC3*, the detection of the mutant allele frequency was especially hampered when the SuperSelective primer mixture was utilized in qPCR as the Cq of the variant could not be properly differentiated from the Cq containing abundant wild-type targets (Supplementary Fig. 2e).

### Validation of low represented variants in normal colonic mucosa using SuperSelective primers by ddPCR

**Figure 3:**
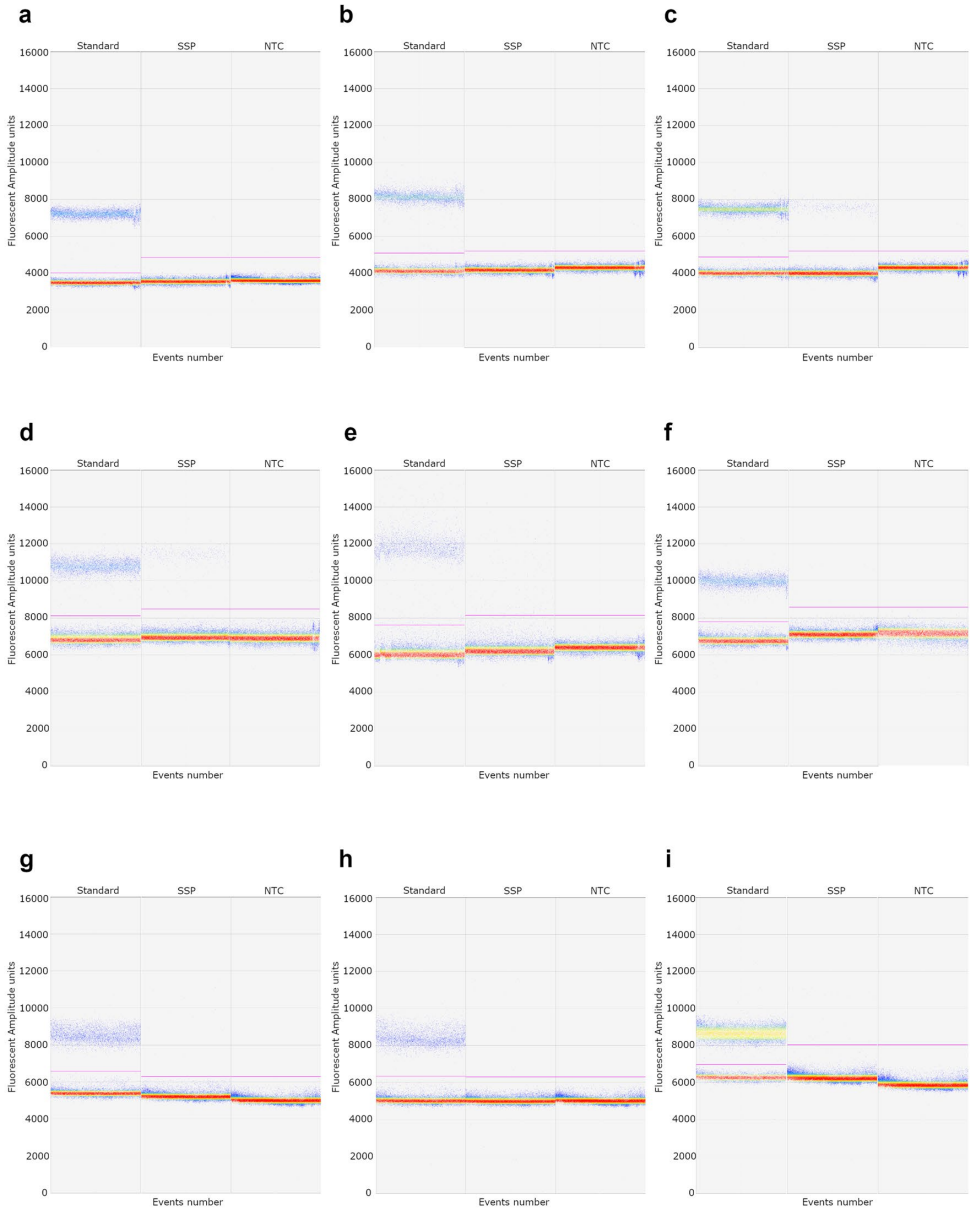
Validation of SNV-associated VAFs by SP-ddPCR in clinical patient samples. Plots show raw data output for fluorescence amplitude. Standard, total copies of the target gene measured by standard primers; *SSP*, quantification of SNV copies by SuperSelective primers; *NTC*, non-template control well containing equivalent amounts of DNA but not the target sequence. (**a**) Surrounding mucosa from *APC* c.4128T>A patient (**b**) Distal mucosa from *APC* c.4128T>A patient (**c**) CRC lesion from *APC* c.4128T>A patient (**d**) Surrounding mucosa from *NRXN3* c.1421G>A patient (**e**) Distal mucosa from *NRXN3* c.1421G>A patient (**f**) CRC lesion from *NRXN3* c.1421G>A patient (**g**) Surrounding mucosa from *NTRK2* c.220G>A patient (**h**) Distal mucosa from *NTRK2* c.220G>A patient (**i**) AAD lesion from *NTRK2* c.220G>A patient.

SuperSelective primers detecting the corresponding somatic variants were used to quantify the VAF by ddPCR in the surrounding and distal normal mucosae, PBLs, and in the colonic lesions from patients with AAD or CRC (Table 3, Fig. 3, Supplementary Fig. 3). First, none of the SNVs were detected in the matched PBL sample when assessed by SP-ddPCR, thus confirming that all variants were somatic. We next validated somatic SNVs c.2626C>T and c.4128T>A affecting *APC* in the CRC samples (VAF of 34.70 ± 0.73% vs 42.96% and 5.61 ± 0.36% vs 6.35% in SP-ddPCR and WES, respectively). In addition, SP-ddPCR confirmed that both *APC* variants were just present in the tumors but neither in the surrounding nor in the distal healthy mucosa (Fig. 3a-c, Supplementary Fig. 3a-c). Then, we sought to confirm the five selected variants identified in the surrounding normal mucosa. Results obtained by ddPCR using custom-designed SuperSelective primers demonstrated the presence of three out of five selected variants in the surrounding normal mucosa, including *NRXN3* c.308G>A, *LAMC3* c.1241G>A, and *ASNA1* c.193C>T (Fig. 3d, Supplementary Fig. 3d,g). The actual quantification indicated that VAFs obtained by SP-ddPCR were lower than those expected from WES, further suggesting that ddPCR using SuperSelective primers can identify VAFs below 10%. In contrast, *NTRK2* c.220G>A and *FABP4* c.105G>A variants in surrounding normal mucosa, which had been detected by WES at 2.70% and 2.74% VAF respectively, showed a VAF of 0.18 ± 0.03% and 0.02 ± 0.01% based on SP-ddPCR (Fig. 3g, Supplementary Fig. 3j). These SP-ddPCR VAFs were below the estimated LODs for the corresponding assays (0.40% for *NTRK2* c.220G>A and 0.10% for *FABP4* c.105G>A). Notably, none of the variants present in the surrounding normal mucosa interrogated by SP-ddPCR was detected in the primary colonic lesions, which agrees with WES data (Fig. 3f,i, Supplementary Fig. 3f,i,l).

**Table 3:** Somatic variants analyzed with SuperSelective primers by ddPCR.

Chromosome	R^†^	A^‡^	Gene Name	HGVS.c	WES total reads	WES variant reads	WES VAF	ddPCR VAF in SM^§^	ddPCR VAF in DM^¶^	ddPCR VAF in CL^††^	ddPCR VAF in PBL^‡‡^
5	T	A	*APC*	c.4128T>A	63	4	6.35%	0.09 ± 0.03%	0.08 ± 0.05%	5.61 ± 0.36%	0.18 ± 0.07%
5	C	T	*APC*	c.2626C>T	142	61	42.96%	0.12 ± 0.05%	0.15 ± 0.01%	34.70 ± 0.73%	0.08 ± 0.03%
9	G	A	*LAMC3*	c.1241G>A	152	11	7.24%	2.51 ± 0.14%	0.51 ± 0.09%	0.14 ± 0.02%	0.07 ± 0.05%
14	G	A	*NRXN3*	c.1421G>A	46	5	10.87%	5.85 ± 0.45%	4.02 ± 1.05%	0.15 ± 0.05%	0.13 ± 0.05%
19	C	T	*ASNA1*	c.193C>T	44	3	6.82%	3.82 ± 0.19%	0.12 ± 0.07%	0.11 ± 0.03%	0.32 ± 0.11%
9	G	A	*NTRK2*	c.220G>A	148	4	2.70%	0.18 ± 0.03%	0.21 ± 0.09%	0.21 ± 0.09%	0.39 ± 0.13%
8	C	T	*FABP4*	c.105G>A	146	4	2.74%	0.02 ± 0.01%	0.10 ± 0.06%	0.06 ± 0.06%	0.09 ± 0.03%

^†^R, Reference.

^‡^A, Alternative.

^§^SM, surrounding mucosa.

^¶^DM, distal mucosa.

^††^CL, colorectal lesion.

^‡‡^PBL, peripheral blood leukocytes.

Finally, we sought to assess whether any of the selected variants occurring in the surrounding normal mucosa were also detected in distal normal mucosa samples by SP-ddPCR. Although we did not have WES data for distal normal mucosa samples to perform a proper comparison, our results indicated that the variant *NRXN3* c.308G>A occurred in the patient’s distal normal mucosa with a SP-ddPCR VAF of 4.02 ± 1.05%, and *LAMC3* c.1241G>A with a VAF of 0.51 ± 0.09%, being both values above the LOD for the corresponding assays (0.14% for *LAMC3* c.1241G>A and 0.19% *NRXN3* c.308G>A) (Fig. 3e, Supplementary Fig. 3e). None of the other variants were identified by SP-ddPCR in the distal normal mucosa (Fig. 3h, Supplementary Fig. 3 h,k).

## Discussion

Quantification of low-represented somatic variants is a key feature in cancer diagnosis and tumor monitoring. Nonetheless, current methodologies utilized for these analyses are complex and costly^12,19,20^. Here, we report the method SP-ddPCR, which combines ddPCR with SuperSelective primers to measure the fractional abundance of somatic mutant alleles in clinical samples.

Previous studies have already shown that ddPCR enabled the quantification of somatic variants with low allelic frequencies^11,21^. However, these ddPCR assays relied on fluorogenic hydrolysis probes, in which several factors such as selectivity, oligonucleotide interactions and baseline signal intensity should be carefully considered during their design^22,23^. SP-ddPCR provides an alternative approach that allows the usage of DNA intercalating dyes for selective amplification of SNVs. Although single-color ddPCR assays have been already utilized to assess SNVs at low VAFs^24^, it is required to design two different allele-specific primers to detect one SNV (wild-type and mutation-specific assays). An advantage of our method is that it only needs the design of one SuperSelective primer that specifically targets the mutant allele, while the wild-type specific amplification is not required.

The selectivity of SuperSelective primers depends on the design of the “3’-foot” and “bridge” sequences. In particular, by exploring different “foot” designs for selective amplification of some of the variants, we observed that the interrogated nucleotide present in the second last position exhibits the best selectivity and accuracy ratios. The second consideration when designing SuperSelective primers is the single-stranded bubble that serves as the “bridge”. The bubble seems to play a role in disrupting the weak mismatched wild-type hybrid in a manner directly proportional to its size^13^. However, it is important not to reduce the abundance of matched mutant hybrids. Thus, the bubble should remain at a minimal size to achieve the amplification of all mutant molecules present in the sample, but sufficient to increase the selectivity of the assay. On the other hand, the 5’-“anchor” sequence, which is also used as standard primer for the target total count, does not provide such selectivity.

In our analysis, estimated LODs and the accurate titration curve of spike-in VAFs were considered acceptable for using SuperSelective primers with ddPCR. Estimated LODs were calculated considering the wild-type background signal. Although further replicates could have been performed to obtain LODs at higher confidence^25^, SP-ddPCR showed LODs comparable to those proposed with hydrolysis-based^12,21^ and other single color alternatives^24^, which are around 0.10% of VAF when evaluating 33 ng of genomic DNA in our assay. The amount of input DNA to guarantee this sensitivity should be specially considered though^26^. Moreover, our results revealed that ddPCR VAF quantifications of in silico samples were more accurate than qPCR, possibly due to the fact that sample partitioning in ddPCR provides absolute quantification. Nevertheless, a recent study suggested that the novel combination of SuperSelective primers with molecular beacons might improve sensitivity when using qPCR assays^18^.

In contrast to WES-derived VAF, read depth using ddPCR is provided based on the quantity of input sample. Thus, a ddPCR reaction with an average of 10,000 target molecules should be equivalent to a 10,000 × coverage in NGS. The proposed approach utilizing SuperSelective primers in ddPCR provides the most precise quantification of SNVs at low VAFs. Results suggested that prioritized variants from WES with VAF below 3% (i.e., *FABP4* c.105G>A and *NTRK2* c.220G>A) were false positive calls from NGS analysis. However, the bioinformatic pipeline applied to the two non-validated variants was less stringent. Indeed, these two SNVs were not called by the intersection between Mutect2 and Strelka. Although the combination of different variant callers might increase the reliability of candidate variants, it considerably limits the list of variants due to the poor consensus between algorithms^27–29^. Our data suggested that the intersection between Mutect2 and Strelka increases confidence in finding true positive calls at low VAFs (i.e., *LAMC3* c.1241G>A, *NRXN3* c.308G>A, and *ASNA1* c.193C>T).

While all three variants confirmed by SP-ddPCR (i.e., *LAMC3* c.1241G>A, *NRXN3* c.308G>A, and *ASNA1* c.193C>T) were present in the surrounding colon mucosa, they were absent in the matched cancerous tissues, advocating a negative selection during tumor evolution. Nonetheless, we found that two out of these three variants (*LAMC3* c.1241G>A and *NRXN3* c.308G>A) were also present in the distal normal mucosa from patients with colorectal carcinoma, although not clonally selected. Intriguingly, these variants had been previously described in colon carcinoma (COSM1256308 and COSM4973279, respectively). We hypothesize that these somatic mutations could have been originated in a common ancestor and have undergone neutral evolution^30–32^.

In summary, this study provides a foundation for the applicability of SP-ddPCR, a rapid, cost-effective ddPCR-derived assay using SuperSelective primers for assessing the presence of rare mutations in cancer patients, thereby contributing to enable personalized medicine.

## Materials and methods

### Primary samples and nucleic acid extraction

A cohort of 48 patients diagnosed with advanced adenoma (AAD), defined by size > 20 mm, or colorectal carcinoma (CRC) were collected between 2013 and 2016. The study was approved by the institutional ethics committee of Hospital General Universitario de Alicante (Ref. CEICPI2013/01), and written informed consent was obtained from all participants in accordance with the Declaration of Helsinki. Both colonic lesions AAD and CRC were collected during colonoscopy or colectomy, respectively. In addition, surrounding (distance to the lesion < 5 cm) and distal normal mucosa (distance to the lesion > 10 cm), as well as peripheral blood leukocytes (PBLs), were obtained from each patient. All tissue samples were conserved in RNAlater (Thermo Fisher Scientific, Waltham, MA, USA) and stored at -80 °C. Normal and neoplastic tissue and PBLs genomic DNA was extracted following the manufacturer’s instructions with Purelink (Thermo Fisher Scientific) and Flexigene (QIAGEN, Venlo, Netherlands) kits, respectively. Double-stranded DNA contained in the elution buffers was quantified using Qubit (Thermo Fisher Scientific) and stored at -20 °C until the time to perform WES or SP-ddPCR.

### Whole-exome sequencing and criteria for SNV selection

Whole-exome sequencing was performed for colonic lesions, surrounding mucosae, and PBLs -used as germline reference-, on a HiSeq 2000 sequencer (Illumina, San Diego, California, USA). Raw data were processed using BWA aligner^33^ with the Human Genome RefSeq GRCh37 followed by standard variant calling by GATK^34^. Somatic variant calling for SNVs and short Indels was performed with Mutect2^35^. Additionally, overlapping with the variant caller Strelka^36^ was performed to reduce false-positive variants in the surrounding normal mucosa from patients with CRC. All callable variants were annotated by SnpEff^37^ and classified into four impact categories: “Low”, “Modifier”, “Moderate”, and “High”. These variants underwent a filtering process that included: (i) cut-off of a minimum number of 10 reads, and (ii) exclusion of variants over-represented in the normal population, defined as those present in the gnomAD database^38^ with an allele frequency > 0.05, considering the highest frequency of all populations. Pathogenic SNVs were determined according to five different predictors. The criteria for each of the five tools to consider a variant as possibly pathogenic were: (i) Polyphen^39^ > 0.85, (ii) LRT^40^ < 0.1, (iii) SIFT^41^ < 0.05, (iv) CADD^42^ > 15, and (v) Mutation Taster^43^ equal to “D” or “D,D”. The Catalogue of Somatic Mutations in Cancer (COSMIC)^44^ was utilized as the reference cancer-related gene list. The selection of variants occurring in surrounding mucosa was performed according to an in-house pipeline based on their potential functional role (Supplementary Fig. 4). Three criteria were considered: 1- variants were classified as “Moderate” and “High” by SnpEff, 2- variants were positive for at least 3 out of 5 pathogenic scores, and 3- variants were reported in COSMIC. Moreover, Integrative Genomics Viewer (IGV)^45^ was used to visually confirm prioritized variants, and manual curation was performed to discard possible false positive calls. In the end, SNVs occurring in healthy colon mucosa from distinct patients and showing allelic frequencies ≤ 10% according to WES analysis were selected as targets for SP-ddPCR detection and quantification.

### Control template generation

To test the accuracy of SuperSelective primers, short control DNA sequences containing the target loci were generated either by cloning the patient sample in plasmid vectors, site-directed mutagenesis or were custom ordered (IDT, Leuven, Belgium). Regardless of how this control sequence was generated, the resulting double-stranded DNA was inserted into the pJET1.2 vector with the CloneJET2.1 PCR Cloning Kit (Thermo Fisher Scientific), and plasmids were isolated using the GeneJET Plasmid Miniprep Kit (Thermo Fisher Scientific). Plasmid templates were used as controls in subsequent steps.

#### Cloning in plasmid vectors

Primer-BLAST^46^ was used to obtain primer pairs for the amplification of 50–80 bp length products containing the referred SNVs. A total of 3.5 ng of DNA (equivalent to 1,000 haploid genomes) from the corresponding patient measured by spectrophotometry was amplified by end-point PCR. The obtained PCR amplicons were cloned in a pJET1.2 vector. DH5α chemically competent cells (Thermo Fisher Scientific) were transformed with the resulting plasmids by heat-shock procedure, plated on selective agar media Luria–Bertani (ampicillin, 60 µg/ml), and incubated at 37 °C overnight. Granted that one colony represents a single molecule template, plasmids from each bacterial colony transformed with each of the variants were isolated using the DNA/RNA/protein solubilization reagent #DCQ100ST (DireCtQuant, Lleida, Spain) and screened by qPCR using SsoAdvanced SYBR Green Supermix (Bio-Rad, Berkeley, CA, USA) following manufacturer’s standard instructions. Next, melting curve analysis was performed to identify differences in the amplicon melting temperature, as a surrogate to detect clones bearing the SNVs. Plasmids were isolated (GeneJET Plasmid Miniprep Kit, Thermo Fisher Scientific) from two colonies of each variant presenting a distinct melting curve corresponding to the wild-type and variant alleles, respectively.

#### Site-directed mutagenesis by PCR

Primers (Supplementary Table 4) were designed to perform site-directed mutagenesis by overlap extension PCR using the previously described wild-type plasmids. In two independent PCR reactions, two fragments of the target sequence were amplified. Each reaction contained one flanking primer that hybridized at one end of the target sequence and one internal primer that hybridized at the variant position and incorporated the mismatched nucleotide in the amplified sequence. These fragments were purified from a 2% agarose gel (GeneJET Gel Extraction kit, Thermo Fisher Scientific). A subsequent extension reaction of five PCR cycles was performed in the absence of the flanking primers to fuse the two resulting fragments followed by a final amplification of the fusion product utilizing the external primers (45 cycles). Finally, synthetic DNA fragments were purified from a 2% agarose gel (GeneJET Gel Extraction kit, Thermo Fisher Scientific).

Bidirectional Sanger sequencing (Stab Vida, Caparica, Portugal) using primers 5'-CGACTCACTATAGGGAGAGCGGC-3' and 5'-AAGAACATCGATTTTCCATGGCAG-3' was performed to validate cloning in plasmids or site-directed mutagenesis products. Data were visualized by Unipro UGENE v.36 (Supplementary Fig. 5).

### SuperSelective primer design

SuperSelective primers (IDT) were designed to include a 5’-“anchor” sequence, a 3’-“foot” sequence containing the interrogated nucleotide that targeted the corresponding SNV site, and a “bridge” sequence between them. The design of SuperSelective primers for the selected variants was performed according to three different strategies for the 3’-“foot”: (i) 6-nt length sequence in which the interrogated nucleotide is the second last (4:1:1), (ii) 7-nt length sequence with an interrogated nucleotide in the second last position (5:1:1), and (iii) 7-nt length sequence containing the interrogated nucleotide in the last position (6:1:0). The “bridge” integrated in the primers was designed to form an asymmetric bubble with the non-complementary sequence of target templates and contained an AT sequence of custom variable length (Fig. 1). Whether the SuperSelective primer was designed as forward or reverse, was decided based on the distance to the interrogated nucleotide and the compatibility of the intermediate sequence to the “bridge” and 3’-“foot”.

Each SuperSelective primer was utilized with the corresponding standard 20-nt length primer pair, specifically designed to present the same melting temperature (Tm). To calculate the SuperSelective primer Tm, only the 5’-“anchor” sequence was used. Both the usual complementary primer and the 5’-“anchor” sequence of the SuperSelective primer –which was also used as the complementary standard primer to measure total target copies– were designed using primer-BLAST with a target annealing temperature of 60 °C to amplify a maximum amplicon length of 100 bp. Standard primer specificity was verified in silico against the Human Genome RefSeq (GRCh37), so that only primer pairs that did not recognize other sequences in the human genome were selected. Measurements of the total number of copies for each gene and the corresponding variants present in the same sample were necessary for quantifying the somatic VAFs. Four different combinations of primer pairs (IDT) were tested to quantify the total target copies present in the ddPCR reaction for *APC* c.2626C>T and *APC* c.4128T>A loci (Supplementary Table 2). In the end, the selected primer pair for measuring the absolute number of copies for the gene in the sample included the 5’-“anchor” sequence as a standard primer in combination with a complementary forward or reverse primer. SuperSelective primer suitability was first tested to ensure the correct amplification of the total somatic molecules while suppressing the wild-type amplification. This was determined over a pure matrix of wild-type or variant-allele plasmids, respectively (Supplementary Table 1). The SuperSelective primers designed for detecting mutations at *APC* (c.2626C>T and c.4128T>A) were compared to three different standard primers, which mimicked the SuperSelective ones, by assessing the VAF in plasmids carrying the SNV (100% VAF) and wild-type plasmids (0% VAF) (Supplementary Table 3). Then, the absence of amplification of wild-type molecules by SuperSelective primers was tested to estimate the limit of detection (LOD) for each SP-ddPCR assay. LOD assessments were performed in at least nine independent SP-ddPCR assays in an excess of an average of 10,000 wild-type plasmid molecules and/or commercially available normal human haploid genomes (Agilent, Santa Clara, CA, USA). LODs were reported as the average VAF, measured in wild-type only sample, plus three times the standard deviation.

### Droplet digital PCR assays

The ddPCR reactions were performed in 20 μl volume containing 10 μl of 2X QX200 ddPCR EvaGreen Supermix (Bio-Rad), 100 nM of each of the mentioned primers, 0.5 μl of FastDigest adequate restriction enzyme (Thermo Fisher Scientific), and the sample at the desired concentration. Dilutions of standard templates were performed using carriers to minimize DNA absorption to polypropylene tubes (VWR, Radnor, PA, USA) and to adjust the number of initial target molecules. Selection of the restriction enzyme was required so that the target sequence was not cut. Next, the reaction mixture was incubated at 37 °C followed by partition and emulsification of the reaction in 70 μl of droplet generation oil for EvaGreen (Bio-Rad) in a QX200 Droplet Generator. The emulsion was transferred to a 96-well plate, and PCR was performed in a thermal cycler (C1000 Deep-well Thermal Cycler, Bio-Rad) with the following cycling conditions: 95 °C for 5 min, 95 °C for 30 s and 60 °C for 60 s for 40 cycles, 4 °C for 5 min, 90 °C for 5 min and cooling to 12 °C for storage before analysis. The number of positive and negative droplets was analyzed using a QX200 Droplet Reader (Bio-Rad). Non-template controls containing all reagents and the corresponding amount of carrier DNA to be equivalent to the tested samples were included in the analysis. The absolute number of copies was calculated with QuantaSoft Analysis Pro (Version 1.0 596, Bio-Rad). ddPCR VAFs were calculated as the mutant copies according to the formula (Fig. 1):

$$\frac{SNV\, copies\, quantified\, by\, SuperSelective\, primer}{total\, gene\, copies\, quantified\, with\, standard\, primers} x 100$$

Results were expressed as mean ± SEM of at least three independent measurements.

### qPCR assays

SuperSelective primers selectivity and quantification obtained by ddPCR was compared to their performance in qPCR using the same sample mixtures from 100 to 0% VAF. The qPCR reactions were also performed in 20 μl volumes, which contained 10 μl of 2X SsoAdvanced SYBR Green Supermix (Bio-Rad) and the same amounts of primers and restriction enzymes as the ddPCR mixtures previously described. After restriction reaction, PCR was thermocycled (Corbett Rotor-Gene 6000, QIAGEN) under the following conditions: 95 °C for 1 min, 95 °C for 5 s and 60 °C for 20 s for 45 cycles, and a melting curve ranging from 65 °C to 95 °C with 0.5 °C increase in each step. Results were expressed as mean ± SEM of at least three independent measurements.

### Supplementary Information

Supplementary Information 1.

Supplementary Figure S1.

Supplementary Figure S2.

Supplementary Figure S3.

Supplementary Figure S4.

Supplementary Figure S5.

## Data Availability

The data that support the findings of this study are available at EGA ID EGAS00001007255 (https://ega-archive.org/studies/EGAS00001007255) and will be shared upon reasonable request.
