# Supplementary material for: Quantification of rare somatic single nucleotide variants by droplet digital PCR using SuperSelective primers
Source: Sci Rep. 2023 Nov 3;13:18997. doi: 10.1038/s41598-023-39874-0 (PMC10624686; doi:10.1038/s41598-023-39874-0)
Supplement: Supplementary file 1 — Supplementary Information 1. [file 41598_2023_39874_MOESM1_ESM.docx]

**SUPPLEMENTARY MATERIAL FOR:**

**Quantification of rare somatic single nucleotide variants by droplet digital PCR using SuperSelective primers**

Verónica Pablo-Fontecha^1,2^, Eva Hernández-Illán^1^, Andrea Reparaz^2^, Elena Asensio^1^, Jordi Morata^3^, Raúl Tonda^3^, Sara Lahoz^1,4^, Carolina Parra^1^, Juan José Lozano^4^, Anabel García-Heredia^5^, Alejandro Martínez-Roca^5^, Sergi Beltran^3^, Francesc Balaguer^1,4^, Rodrigo Jover^5^, Antoni Castells^1,4^, Ramon Trullàs^2,6^, Petar Podlesniy^2^, Jordi Camps*^1,4,7^

1 Translational Colorectal Cancer Genomics, Gastrointestinal and Pancreatic Oncology Team, Institut D'Investigacions Biomèdiques August Pi i Sunyer (IDIBAPS), Hospital Clínic de Barcelona, 08036, Barcelona, Spain

2 Centro de Investigación Biomédica en Red sobre Enfermedades Neurodegenerativas (CIBERNED), 28029, Madrid, Spain

3 CNAG-CRG, Centre for Genomic Regulation (CRG), Barcelona Institute of Science and Technology (BIST), 08028, Barcelona, Spain

4 Centro de Investigación Biomédica en Red de Enfermedades Hepáticas y Digestivas (CIBEREHD), 28029, Madrid, Spain

5 Servicio de Medicina Digestiva, Hospital General Universitario de Alicante, Instituto de Investigación Sanitaria y Biomédica de Alicante (ISABIAL), 03010, Alicante, Spain

6 Neurobiology Unit, Institut d'Investigacions Biomèdiques de Barcelona (IIBB-CSIC), Institut d'Investigacions Biomèdiques August Pi i Sunyer (IDIBAPS), 08036 Barcelona, Spain

7 Unitat de Biologia Cel·lular i Genètica Mèdica, Departament de Biologia Cel·lular, Fisiologia i Immunologia, Facultat de Medicina, Universitat Autònoma de Barcelona, Bellaterra 08193, Spain

**SUPPLEMENTARY TABLES**

**Supplementary Table 1**. SuperSelective sequences tested during primer design development.

| Target gene (HGVS.c) | Super-Selective primer strategy | Super-Selective primer (sequence 5'-3') | ddPCR SNV quantification in 100% SNV matrix | ddPCR SNV quantification in 100% wild-type matrix |
| --- | --- | --- | --- | --- |
| *APC (*c.4128T>A*)* | 21-10/9-4:1:1 | GTGCTCAGACACCCAAAAGTC -ATATATATAT-ACTA**A**G | 4.62 % | 0.02 % |
| *APC (*c.4128T>A) | 21-6/9-4:1:1 | GTGCTCAGACACCCAAAAGTC -ATATAT-ACTA**A**G | 31.50 % | Undetectable |
| *APC (*c.4128T>A) | 21-10/8-5:1:1 | GTGCTCAGACACCCAAAAGTC -ATATATATAT-CACTA**A**G | 63.10 % | Undetectable |
| *APC (*c.4128T>A) | 21-10/7-6:1:0 | GTGCTCAGACACCCAAAAGTC -ATATATATAT-ACACTA**A** | 49.80 % | 0.03 % |
| *APC (*c.4128T>A) | 21-7/8-5:1:1 | GTGCTCAGACACCCAAAAGTC -ATATATA-CACTA**A**G | 93.56 % | 0.10 % |
| *APC* (c.2626C>T) | 15-18/10-4:1:1 | GGCTGCAGTGGTGGA-ATATATATATATATATAT-CCTC**A**C | Undetectable | Undetectable |
| *APC* (c.2626C>T) | 15-18/9-5:1:1 | GGCTGCAGTGGTGGA-ATATATATATATATATAT-ACCTC**A**C | Undetectable | Undetectable |
| *APC* (c.2626C>T) | 15-18/8-6:1:0 | GGCTGCAGTGGTGGA-ATATATATATATATATAT-AACCTC**A** | Undetectable | Undetectable |
| *APC* (c.2626C>T) | 15-10/10-4:1:1 | GGCTGCAGTGGTGGA- ACGTGATCCC -CCTC**A**C | Undetectable | Undetectable |
| *APC* (c.2626C>T) | 18-8/7-4:1:1 | GGCTGCAGTGGTGGAGAT- ATATATAT -CCTC**A**C | 84.15 % | 0.08 % |
| *LAMC3* (c.1241G>A) | 22-8/6-4:1:1 | CCTCACTGAGCGAGTGGAACCC-ATATATAT-ACAG**T**G | Undetectable | Undetectable |
| *LAMC3*  (c.1241G>A) | 19-8/6-4:1:1 | CACTGAGCGAGTGGAACCC-ATATATAT-ACAG**T**G | 62.35 % | 0.02 % |
| *NRXN3* (c.1421G>A) | 21-8/5-4:1:1 | AACGTATGGGCTCCATCTCCT-ATATATAT-TTCC**A**C | 96.33 % | 0.10 % |
| *ASNA1* (c.193C>T) | 22-8/5-4:1:1 | GGGTCTGTGGAGATGATCAGAA-ATATATAT-TCAC**A**C | 96.64 % | 0.36 % |
| *ASNA1* (c.193C>T) | 22-8/4-5:1:1 | GGGTCTGTGGAGATGATCAGAA-ATATATAT-CTCAC**A**C | 103.44 % | 43.51 % |
| *ASNA1*(c.193C>T) | 22-8/3-6:1:0 | GGGTCTGTGGAGATGATCAGAA-ATATATAT-TCTCAC**A** | 96.51 % | 0.91 % |
| *ASNA1*(c.193C>T) | 22-4/5-4:1:1 | GGGTCTGTGGAGATGATCAGAA-TATA-TCAC**A**C | 109.03 % | 86.78 % |
| *ASNA1*(c.193C>T) | 22-5/5-4:1:1 | GGGTCTGTGGAGATGATCAGAA-TATAT-TCAC**A**C | 92.78 % | 31.36 % |
| *ASNA1*(c.193C>T) | 22-6/5-4:1:1 | GGGTCTGTGGAGATGATCAGAA-TATATA-TCAC**A**C | 97.67 % | 22.34 % |
| *ASNA1*(c.193C>T) | 22-7/5-4:1:1 | GGGTCTGTGGAGATGATCAGAA-TATATAT-TCAC**A**C | 89.35 % | 2.31 % |
| *ASNA1*(c.193C>T) | 22-8/5-4:1:1 | GGGTCTGTGGAGATGATCAGAA-ATATATAT-TCAC**A** | 96.64 % | 0.56 % |
| *ASNA1*(c.193C>T) | 22-9/5-4:1:0 | GGGTCTGTGGAGATGATCAGAA-TATATATAT-TCAC**A**C | 85.14 % | 0.65 % |
| *ASNA1*(c.193C>T) | 22-10/5(A)-4:1:1 | GGGTCTGTGGAGATGATCAGAA-ATATATATAA-TCAC**A**C | 90.46 % | 0.46 % |
| *ASNA1*(c.193C>T) | 22-10/5(T)-4:1:1 | GGGTCTGTGGAGATGATCAGAA-ATATATATAT-TCAC**A**C | 61.07 % | 0.31 % |
| *ASNA1*(c.193C>T) | 22-10/5-4:1:1 | GGGTCTGTGGAGATGATCAGAA-ATATATATATA-TCAC**A**C | 94.42 % | 0.19 % |
| *NTRK2* (c.220G>A) | 25-8/4-4:1:1 | TCTTCGTTGATGATTTCTAACCTTT - ATATATAT - TTTG**T**G | Undetectable | Undetectable |
| *NTRK2* (c.220G>A) | 28-6/4-5:1:1 | CATCTTCGTTGATGATTTCTAACCTTTT - A TATAT - GTTTG**T**G | 89.74 % | 0.05 % |
| *FABP4* (c.105G>A) | 17-8/5-4:1:1 | TTTGCCACCAGGAAAGT - ATATATAT - CATG**A**C | Undetectable | Undetectable |
| *FABP4* (c.105G>A) | 20-6/5-4:1:1 | GCTTTGCCACCAGGAAAGTG - TATATA - CATG**A**C | 77.11 % | 0.03 % |
| In bold are indicated the positions of the interrogated nucleotides. | | | | |

**Supplementary Table 2**. Standard primers designed to quantify total copies of the SNV locus.

| Target gene (HGVS.c) | Standard primer (sequence 5’-3’) | Primer direction | Tm (°C) |
| --- | --- | --- | --- |
| *APC* (c.4128T>A) | GTGCTCAGACACCCAAAAGTC | Forward | 59.40 |
| *APC* (c.4128T>A) | TCCCTCCAAAAGTGGTGCTC | Forward | 59.89 |
| *APC* (c.4128T>A) | ATCTGCTAAACATGAGTGGGGTC | Reverse | 60.37 |
| *APC* (c.4128T>A) | ACATGAGTGGGGTCTCCTGA | Reverse | 59.88 |
| *APC* (c.2626C>T) | GCAACAGAAAATCCAGGAACTTCT | Forward | 59.72 |
| *APC* (c.2626C>T) | AACTACCATCCAGCAACAGAAAATC | Forward | 59.82 |
| *APC* (c.2626C>T) | GGCTGCAGTGGTGGAGAT | Reverse | 59.33 |
| *APC* (c.2626C>T) | CTTCCATGACTTTGGCAATCTGG | Reverse | 60.12 |

**Supplementary Table 3**. Standard primers designed to selectively quantify SNV copies.

| Target gene (HGVS.c) | Standard primer strategy | Standard primer (sequence 5'-3') | ddPCR SNV quantification in 100% SNV matrix | ddPCR SNV quantification in 100% wild-type matrix |
| --- | --- | --- | --- | --- |
| *APC* (c.4128T>A) | 23-1:0 | CCCAAAAGTCCACCTGAACACTA**A** | 109.48 | 70.66 |
| *APC* (c.4128T>A) | 22-1:1 | CCAAAAGTCCACCTGAACACTA**A**G | 104.01 | 93.76 |
| *APC* (c.4128T>A) | 34-1:1 | GTGCTCAGACACCCAAAAGTCCACCTGAACACTA**A**G | 103.70 | 100.51 |
| *APC* (c.2626C>T) | 20-1:0 | GGTGGAGATCTGCAAACCTC**A** | 95.85 | 93.79 |
| *APC* (c.2626C>T) | 19-1:1 | GTGGAGATCTGCAAACCTC**A**C | 99.43 | 92.59 |
| *APC* (c.2626C>T) | 29-1:1 | GGCTGCAGTGGTGGAGATCTGCAAACCTC**A**C | 98.64 | 96.61 |
| In bold are indicated the positions of the interrogated nucleotides. | | | | |

**Supplementary Table 4**. Site-directed mutagenesis primer sequences.

| Primer | Tm | Primer sequence (5’-3’) |
| --- | --- | --- |
| FABP4-Fmut | 60,32 | TAGGTTTGGTCATGCCAGCC |
| FABP4-Rmut | 60,32 | GGCTGGCATGACCAAACCTA |
| NTRK2-Fmut | 57,87 | TGTTACAGTTTCATCACAAACCAG |
| NTRK2-Rmut | 57,87 | CTGGTTTGTGATGAAACTGTA |
| LAMC3-Fmut | 62,69 | TGGAAGTGTGACCACTGTCTGC |
| LAMC3-Rmut | 62,69 | GCAGACAGTGGTCACACTTCCA |

**SUPPLEMENTARY FIGURE LEGENDS**

**Supplementary Figure 1**. Comparison of *APC* c.2626C>T and *APC* c.4128T>A loci quantification by using four different standard primer combinations for each target.

**Supplementary Figure 2**. Comparison of SuperSelective primers variant allele frequency (VAF) quantification accuracy in ddPCR vs qPCR. Selective quantification of each single nucleotide variant (SNV) in the same synthetic plasmid mixtures simulating VAFs from 100 to 0.0% in ddPCR compared to qPCR using the same set of primers. (a) *APC* c.2626C>T (b) *APC* c.4128T>A (c) *FABP4* c.105G>A (d) *NTRK2* c.220G>A (e) *LAMC3* c.1241G>A (f) *NRXN3* c.308G>A (g) *ASNA1* c.193C>T.

**Supplementary Figure 3.** Validation of variant allele frequencies (VAFs) of single nucleotide variants (SNVs) by SP-ddPCR (SuperSelective primers in droplet digital PCR) in clinical patient samples. Plots show raw data output for 1-D fluorescence amplitude. *Standard* exhibits quantification of total copies of the target gene per well measured by standard primers; *SSP*, quantification of SNV copies by SuperSelective primers; *NTC*, non-template control well with containing equivalent amounts of DNA but not the target sequence (a) Surrounding mucosa from *APC* c.2626C>T patient (b) Distal mucosa from *APC* c.2626C>T patient (c) CRC lesion from *APC* c.2626C>T patient (d) Surrounding mucosa from *LAMC3* c.1241G>A patient (e) Distal mucosa from *LAMC3* c.1241G>A patient (f) CRC lesion from *LAMC3* c.1241G>A patient (g) Surrounding mucosa from *ASNA1* c.193C>T patient (h) Distal mucosa from *ASNA1* c.193C>T patient (i) CRC lesion from *ASNA1* c.193C>T patient (j) Surrounding mucosa from *FABP4* c.105G>A patient (k) Distal mucosa from *FABP4* c.105G>A patient (l) CRC lesion from *FABP4* c.105G>A patient.

**Supplementary Figure 4**. Bioinformatic pipeline for variant prioritization in advanced adenoma (AAD) and colorectal carcinoma (CRC) normal surrounding mucosa. The mean number of variants per patient (x̅) filtered through the first two steps and the total number of variants (n) passing the following ones are shown.

**Supplementary Figure 5.** Wild-type and mutant template sequences generated by cloning or site-directed mutagenesis.
