# Supplementary figures and images for: Quantification of rare somatic single nucleotide variants by droplet digital PCR using SuperSelective primers
Source: Sci Rep. 2023 Nov 3;13:18997. doi: 10.1038/s41598-023-39874-0 (PMC10624686; doi:10.1038/s41598-023-39874-0)

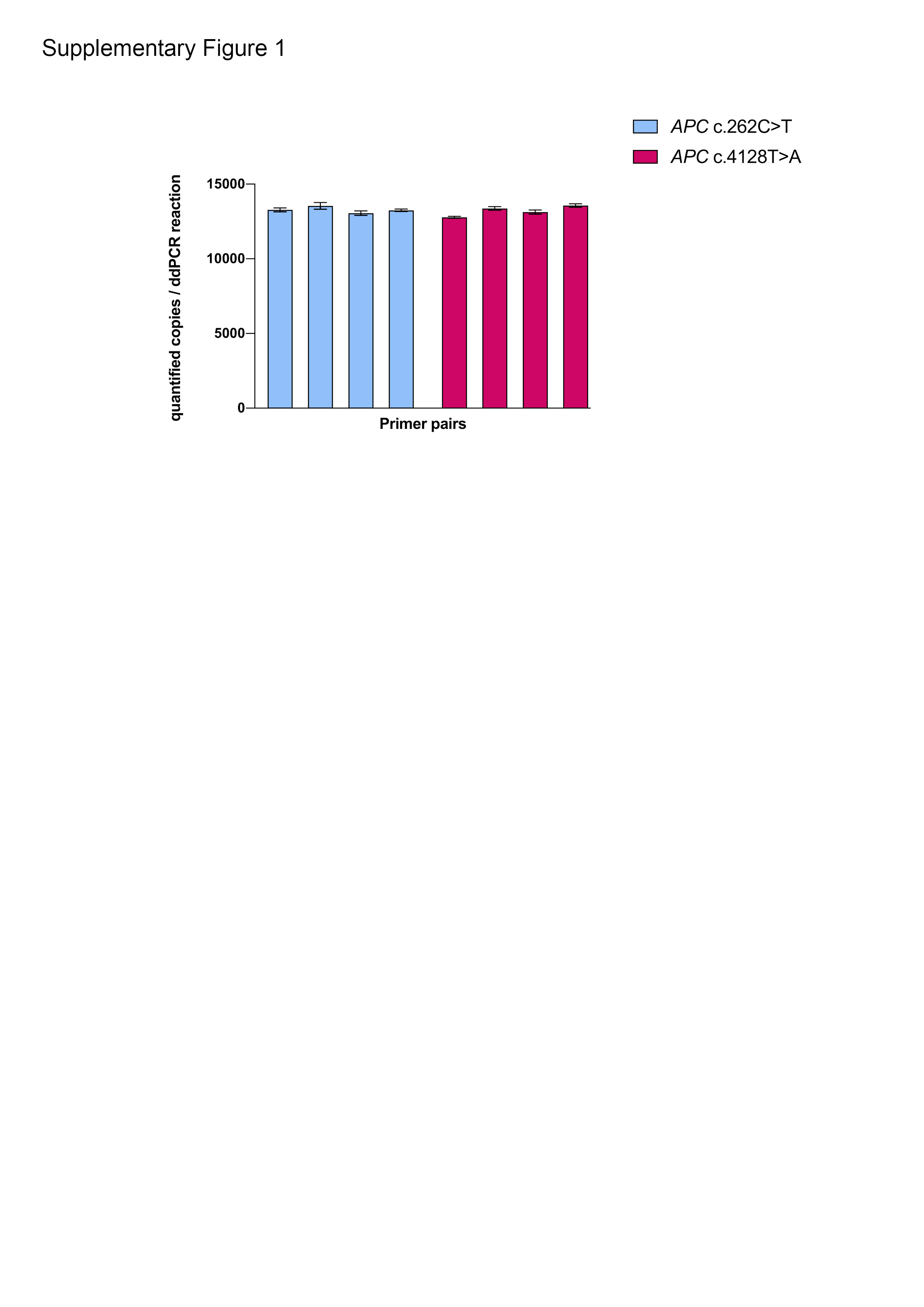

Supplement: Supplementary file 2 — Supplementary Figure S1. [file 41598_2023_39874_MOESM2_ESM.tif]

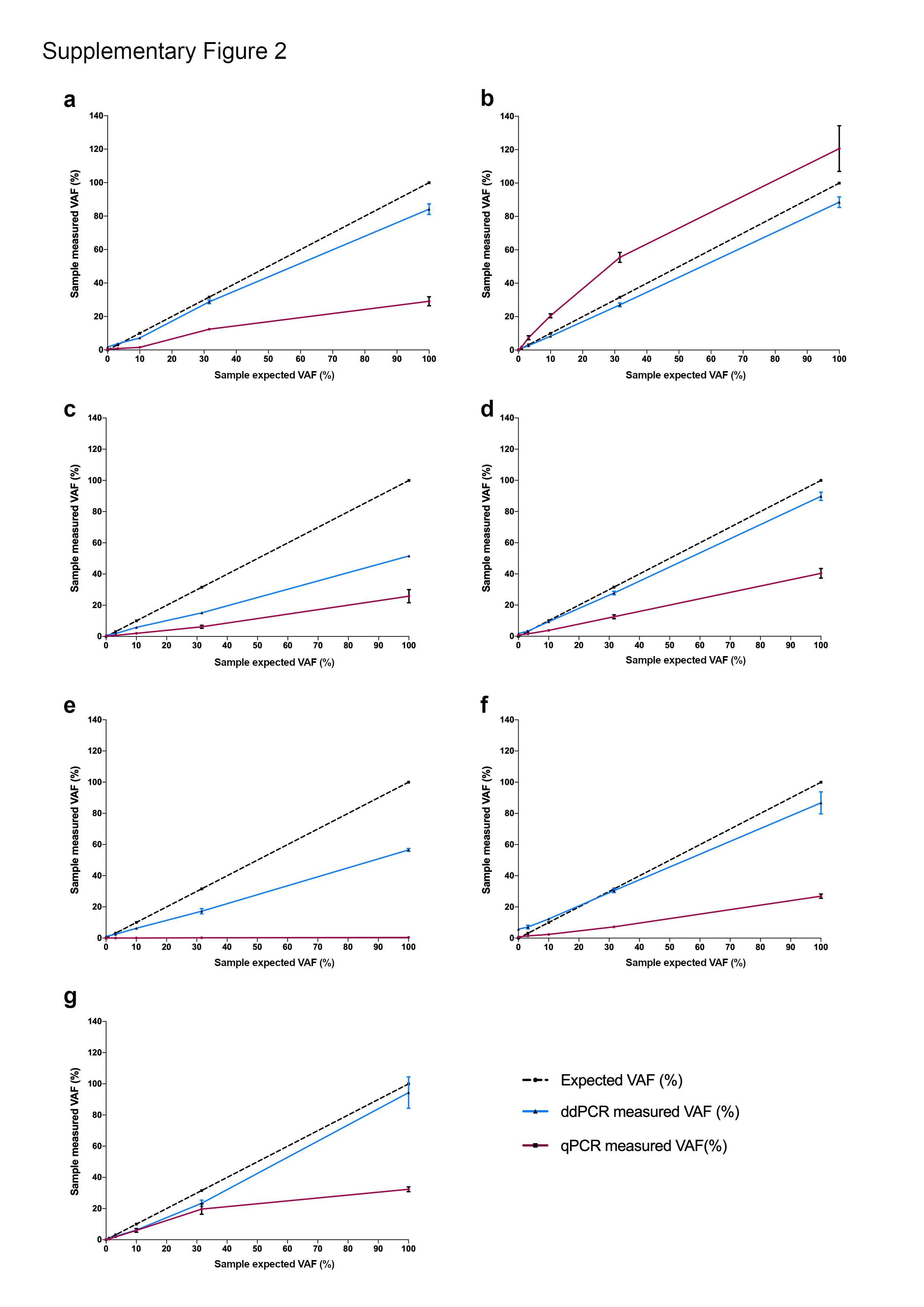

Supplement: Supplementary file 3 — Supplementary Figure S2. [file 41598_2023_39874_MOESM3_ESM.tif]

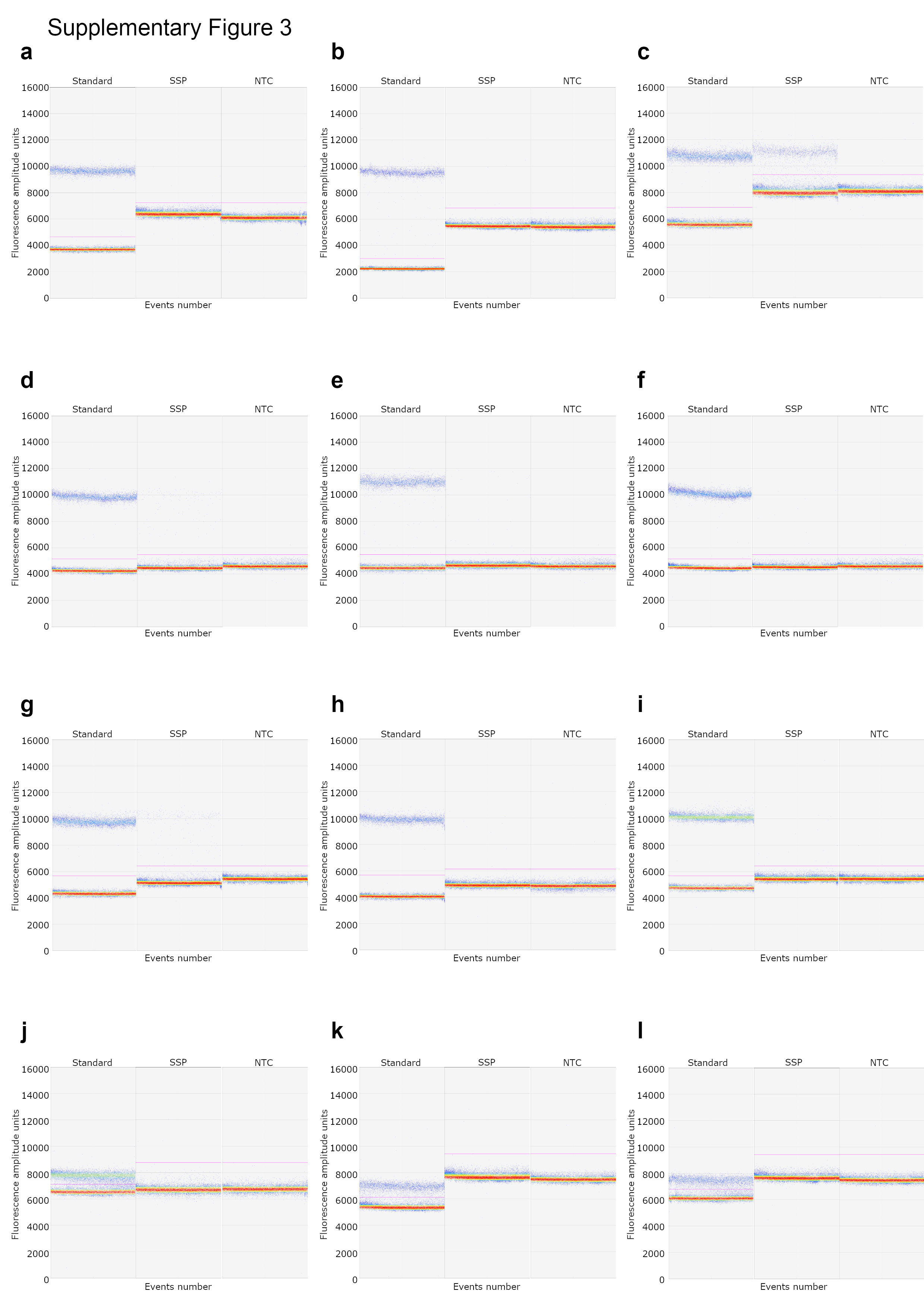

Supplement: Supplementary file 4 — Supplementary Figure S3. [file 41598_2023_39874_MOESM4_ESM.tif]

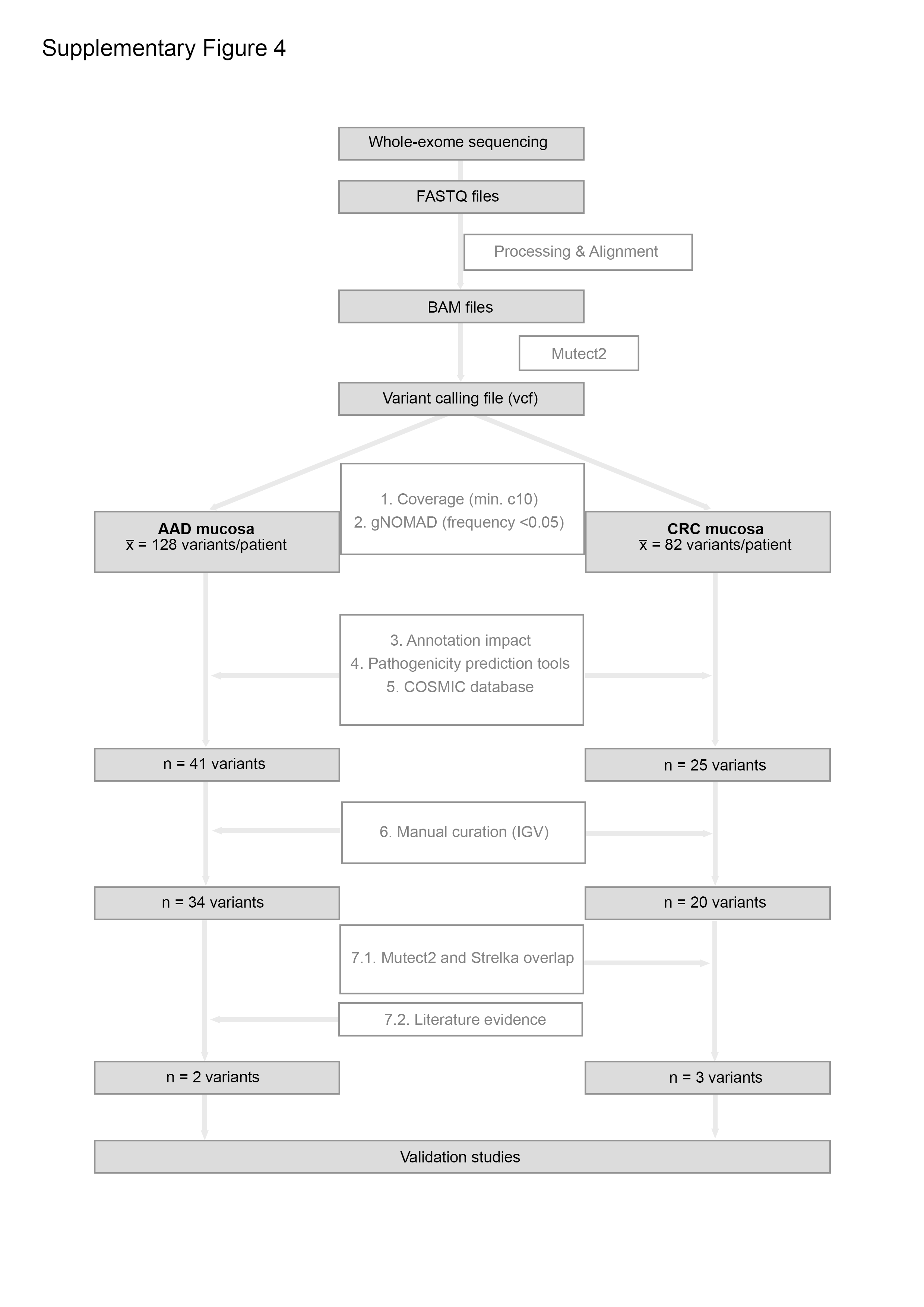

Supplement: Supplementary file 5 — Supplementary Figure S4. [file 41598_2023_39874_MOESM5_ESM.tif]

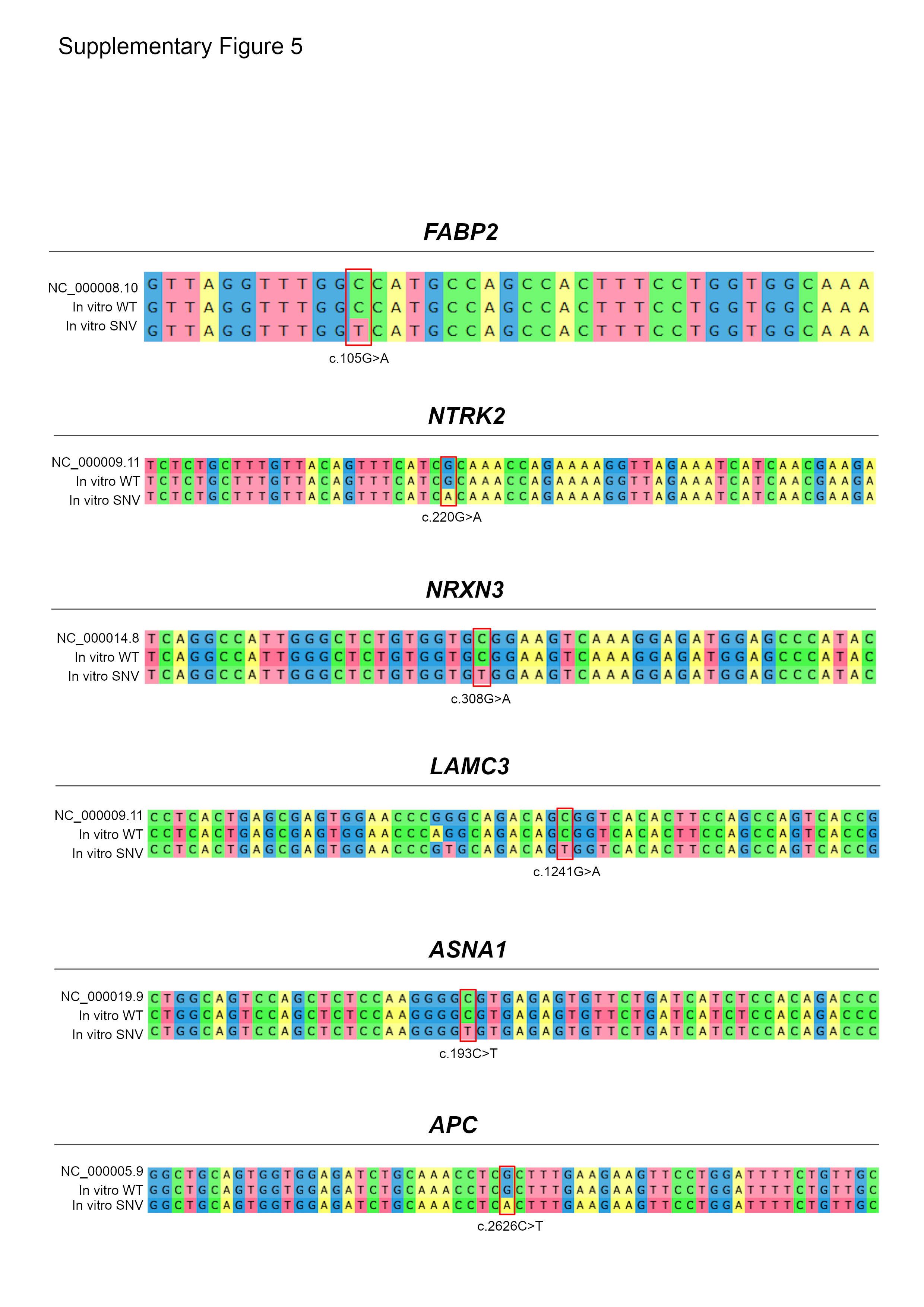

Supplement: Supplementary file 6 — Supplementary Figure S5. [file 41598_2023_39874_MOESM6_ESM.tif]
